# Incompatibilities Involving Yeast Mismatch Repair Genes: A Role for Genetic Modifiers and Implications for Disease Penetrance and Variation in Genomic Mutation Rates

**DOI:** 10.1371/journal.pgen.1000103

**Published:** 2008-06-20

**Authors:** Ann Demogines, Alex Wong, Charles Aquadro, Eric Alani

**Affiliations:** Department of Molecular Biology and Genetics, Cornell University, Ithaca, New York, United States of America; National Institute of Diabetes and Digestive and Kidney Diseases, United States of America

## Abstract

Genetic background effects underlie the penetrance of most genetically determined phenotypes, including human diseases. To explore how such effects can modify a mutant phenotype in a genetically tractable system, we examined an incompatibility involving the *MLH1* and *PMS1* mismatch repair genes using a large population sample of geographically and ecologically diverse *Saccharomyces cerevisiae* strains. The mismatch repair incompatibility segregates into naturally occurring yeast strains, with no strain bearing the deleterious combination. In assays measuring the mutator phenotype conferred by different combinations of *MLH1* and *PMS1* from these strains, we observed a mutator phenotype only in combinations predicted to be incompatible. Surprisingly, intragenic modifiers could be mapped that specifically altered the strength of the incompatibility over a 20-fold range. Together, these observations provide a powerful model in which to understand the basis of disease penetrance and how such genetic variation, created through mating, could result in new mutations that could be the raw material of adaptive evolution in yeast populations.

## Author Summary

For many common afflictions, it is difficult to map disease-associated loci because multiple loci are involved, with some loci playing greater roles than others. To explore how complex interactions can contribute to disease, we examined an incompatibility involving the MLH1 and PMS1 DNA mismatch repair proteins in baker's yeast. In our system, an incompatibility is defined as a defect occurring when specific combinations of MLH1 and PMS1 proteins obtained from different baker's yeast strains are tested for function. We identified amino acid differences at only one site in each protein that contributed to this incompatibility. We also showed that amino acid differences that could cause such an incompatibility are found in strains collected from across the globe. No strain contained the incompatible MLH1-PMS1 combination, indicating that it was likely to be deleterious. When such a combination was created in the laboratory, we could detect a wide range of defects that were under the control of genetic modifiers. These observations provide a powerful model in which to understand the basis of disease penetrance and how segregation of defects in mismatch repair may allow for rapid yet reversible changes in genomic mutation rates that can help yeast adapt to changing or novel environments.

## Introduction

The scientific literature contains numerous examples in which alleles confer more severe phenotypes in one genetic background relative to another (e.g. [1],[2]). Such effects are thought to be due to DNA sequence differences at multiple genetic loci that lead to molecular incompatibilities between gene products or between gene products and cis-acting regulatory sequences that function in specific pathways. Identifying such incompatibilities in a genetically tractable system is of great interest because they provide testable models to study disease penetrance, defined as the proportion of individuals that carry disease-linked alleles that also display the disease phenotype (e.g. [3],[4],[5]).

We have been studying molecular incompatibilities that relate to DNA mismatch repair (MMR). MMR improves the fidelity of DNA replication ∼1000 fold by repairing mismatches that arise during DNA replication. In addition, factors that act in MMR play an important role in preventing interspecific gene transfer by rejecting recombination intermediates containing multiple DNA mismatches [6],[7],[8]. In *E. coli*, DNA mispairs are recognized by a homodimer of the MutS protein, which then recruits the MutL homodimer. MutL coordinates recognition of a mispair with downstream excision repair activities. In yeast and other eukaryotes, multiple MutS and MutL homologues (MSH and MLH) have evolved to function as heterodimers with specialized functions in DNA repair and recombination. The primary MLH heterodimer in yeast MMR is MLH1-PMS1 (reviewed in [8]). In humans, mutations in *MSH* and *MLH* genes have been implicated in hereditary non-polyposis colorectal cancer (HNPCC), a dominantly inherited syndrome. Recent studies in humans and yeast suggest that genetic background can play an important role in the level of pathogenicity conferred by MMR alleles [9],[10].

Previously our laboratories (Heck et al. [11]) examined *S. cerevisiae* progeny created by mating SK1 and S288c strains that are ∼0.7% sequence divergent. We identified single amino acid polymorphisms in *MLH1* and *PMS1* that, in combination, lead to an elevation in mutation rate and a generalized reduction in long-term fitness. These reductions in fitness are analogous to those seen for interactions thought to be the basis for hybrid incompatibility between incipient or established species [12]–[16]. Heck et al. [11] proposed a negative-epistasis model in which the S288c and SK1 strains diverged from an ancestral population. When the strains are crossed, an incompatible combination is generated at a 25% frequency.

Several critical questions emerged from this analysis with respect to the negative epistatic *MLH1-PMS1* interactions resulting from hybrid incompatibility. First, is there evidence of genetic divergence at the *PMS1* and *MLH1* loci, as predicted if the incompatible alleles arose in isolation? Second, do the amino acid polymorphisms isolated by Heck et al. [11] confer incompatibility regardless of allelic background? Third, do intragenic modifiers of the incompatibility segregate in natural populations? Because each of the above questions is best answered using a large sample of *PMS1* and *MLH1* alleles, we obtained the DNA sequence of the *MLH1* and *PMS1* genes from 68 geographically and ecologically diverse strains of *S. cerevisiae.* These strains were genotyped for the single amino acid polymorphisms in *MLH1* and *PMS1* that conferred a mutator phenotype in S288c-SK1 hybrids. We found that none of the strains sampled contained the incompatible combination. Furthermore, a mutator phenotype was only observed in *MLH1-PMS1* combinations that contained the single polymorphisms underlying the S288c-SK1 incompatibility. To our surprise the mutator phenotype observed in this set of combinations varied over a 20-fold range. In contrast, all possible compatible combinations showed mutation rates similar to those seen in natural populations. We mapped the variation/genetic modifiers seen in the incompatible combinations to specific polymorphisms within *MLH1* and *PMS1*. This work demonstrates a mechanism by which yeast can rapidly and reversibly elevate the genomic mutation rate in natural populations. It also illustrates a model for disease penetrance that may explain the basis of some HNPCC-like cancers displaying atypical inheritance [9].

## Results

### The Model for MLH1-PMS1 Hybrid Incompatibility Holds True Regardless of Allelic Origin

Heck et al. [11] identified one site each in MLH1 (aa 761, Asp in S288c, Gly in SK1) and PMS1 (aa 818/822, Arg in S288c, Lys in SK1) that accounted for the MMR defects observed in progeny derived from crossing S288c and SK1 strains. This analysis indicated that only the MLH1-Asp, PMS1-Lys combination conferred the mutator phenotype. Using a limited sampling of *Saccharomyces cerevisiae* strains and closely-related *Saccharomyces* species, Heck et al. [11] inferred that MLH1-Gly, PMS1-Arg was the predicted ancestral combination. None of the 14 strains in this small sample contained the MLH1-Asp, PMS1-Lys combination predicted to be a mutator, despite DNA sequence analysis suggesting that recombination had occurred between strains that could generate this combination. An explanation for this observation, which was supported by spore viability after mutation accumulation in bottleneck growth studies, is that the MLH1-Asp, PMS1-Lys combination carries a long-term fitness cost.

**Figure 1:**
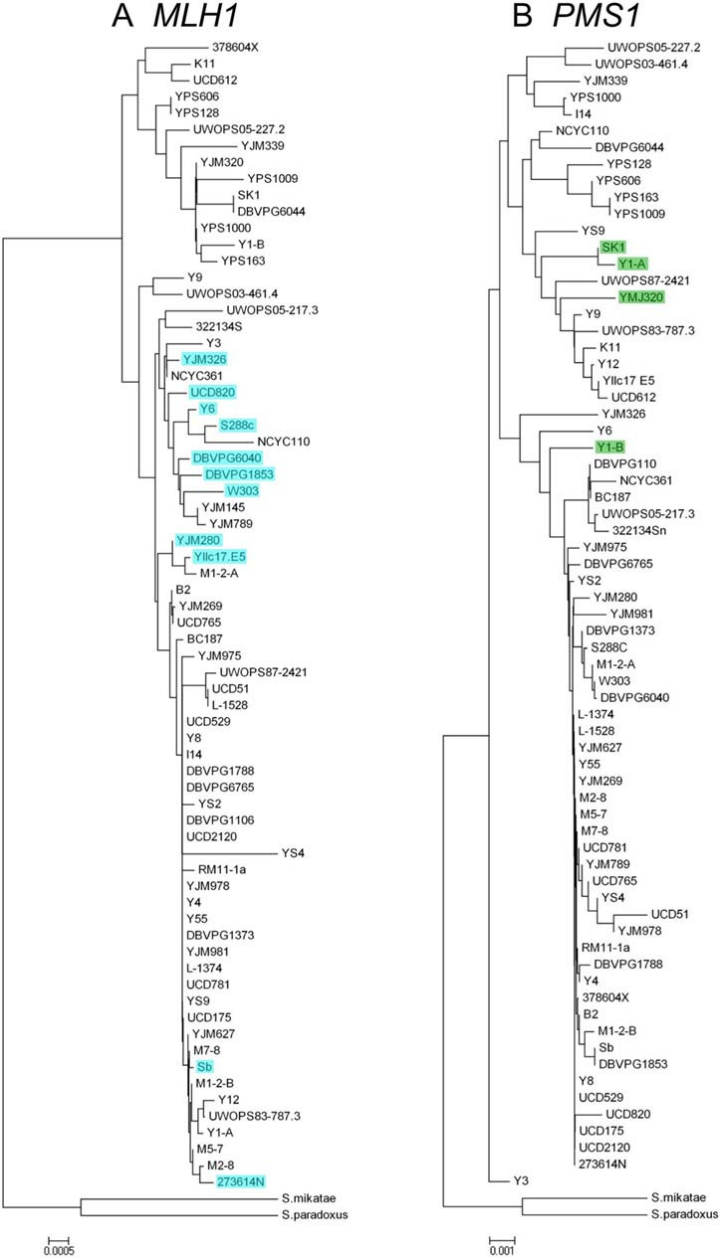
Phylogenetic relationships amongst *MLH1* and *PMS1* gene sequences. (A) Neighbor-joining tree of *MLH1* gene sequences for all 68 strains. The nucleotide site responsible for the MLH1 polymorphism at amino acid 761 was excluded from the phylogenetic reconstruction. The strains that are shaded in blue all contain the MLH1-D761 polymorphism originally identified in the S288c strain. Close relationships amongst seven strains carrying the MLH1-D761 polymorphism suggest a common origin for this allele, although extensive recombination or multiple origins must be invoked to explain more distant relationships to four additional D761 strains. Terminal branches leading to the two outgroup species *S. mikatae* and *S. paradoxus*, as well as the internal branch leading to the outgroups, are not drawn to scale. (B) Neighbor-joining tree of *PMS1* gene sequences for 67 strains. The nucleotide site responsible for the PMS1 polymorphism at amino acid 822 was excluded from the phylogenetic reconstruction. The strains that are shaded in green all contain the PMS1-K822 polymorphism originally identified in the SK1 strain. The low frequency of this allele makes inferences regarding its origin difficult. See Materials and Methods for details. The blue/green color scheme is maintained in Figure 2, Table S1, and Table S2.

Here we examined 68 yeast strains to measure the frequency of the incompatibility alleles (Figure 1, Table 1). The selected strains included natural, clinical, and domesticated stocks from a variety of collections, including the strains compiled by Justin Fay [17], strains currently being sequenced by the Saccharomyces Genome Resequencing Project at the Sanger Institute (http://www.sanger.ac.uk/Teams/Team71/durbin/sgrp/), the recently sequenced vineyard isolate RM11-1a (http://www.broad.mit.edu/annotation/fungi/comp_yeasts/), and the clinical strain YJM789 [18]. For each of the strains listed in Table 1, *MLH1* and *PMS1* were amplified and the sequence surrounding the S288c-SK1 incompatibility defined above was determined (except YJM789, sequence solely from public database). Additional sequencing or publicly available sequence was used to determine the additional SNPs located in the *MLH1* and *PMS1* open reading frames as well as downstream sequence (Table S1 and Table S2). Neighbor-joining trees were created for *MLH1* and *PMS1* using the compiled sequence data for *Saccharomyces cerevisiae* and closely related *Saccharomyces* species (Figure 1A and B). A total of 72 polymorphic sites were found in and around *MLH1*, with an average pairwise nucleotide diversity (π) of 0.002. 134 polymorphic sites were identified in and around *PMS1* (π = 0.0039). Sequencing of *MLH1* and *PMS1* revealed a pattern similar to that seen by Fay and Benavides [19]; the domesticated strains tended to group, consistent with the idea that they are derived from a subset of wild-type yeast.

**Table 1:** *S. cerevisiae* strains analyzed in this study.

Strain	Collection	Geographic	Source	*MLH1*	*PMS1*	Class
S288c	-	California, USA	Rotting fig	S	S	C
SB (S.boulardii)	Fay	Indonesia	Nature (lychee fruit)	S	S	C
Y6 (NRRL yb1952)	Fay	French Guiana	NA	S	S	C
UCD820 (UC8)	Fay	South Africa	Vineyard	S	S	C
W303	Sanger	Unknown	Rodney Rothstein*	R	R	C
273614N	Sanger	RVI, Newcastle UK	Clinical isolate (Fecal)	S	S	C
YIIc17_E5	Sanger	Sauternes, France	Wine	S	S	C
DBVPG1853	Sanger	Ethiopia	White Tecc	S	S	C
DBVPG6040	Sanger	Netherland	Fermenting fruit juice	S	S	C
YJM269	McCusker	NA	Fermentation (apple juice)	S	R	C
YJM280	McCusker	United States	Clinical	S	R	C
SK1	Sanger	USA	Soil	R	R	K
YJM320	McCusker	United States	Clinical	S	R	K
Y1 (NRRL y390)	Fay	NA	Nature (mushroom)	S	S	K
Y4 (NRRL y1532)	Fay	Indonesia	Nature (fruit)	S	S	A
YPS163	Fay	Pennsylvania, USA	Nature (oak exudate)	S	S	A
YPS1000	Fay	New Jersey	Nature (oak exudate)	S	S	A
YPS1009	Fay	New Jersey	Nature (oak exudate)	S	S	A
YJM326	McCusker	United States	Clinical	S	R	A
YJM145	McCusker	Missouri, USA	Clinical	S	R	A
YJM627	McCusker	France	NA	S	R	A
YJM339	McCusker	United States	Clinical	S	R	A
M1-2	Townsend	Italy	Vineyard	S	S	A
M2-8	Townsend	Italy	Vineyard	S	S	A
M5-7	Townsend	Italy	Vineyard	S	S	A
M7-8	Townsend	Italy	Vineyard	S	S	A
I14	Fay	Italy	Vineyard (soil)	S	S	A
UCD612 (UC5)	Fay	Kurashi, Japan	Sake	S	S	A
UCD2120 (UC10)	Fay	California, USA	Vineyard	S	S	A
UCD51 (UC1)	Fay	France	Vineyard	S	S	A
UCD529 (UC4)	Fay	Germany	Vineyard	S	S	A
UCD781 (UC7)	Fay	Switzerland	Vineyard	S	S	A
B2 (levuline ALS)	Fay	NA	Vineyard (commercial)	S	S	A
Y8 (NRRL y2411)	Fay	Turkey	Vineyard	S	S	A
UCD175 (UC2)	Fay	Sicily, Italy	Vineyard	S	S	A
Y3 (NRRL y1438)	Fay	Africa	Fermentation (palm wine)	S	S	A
UCD765 (UC6)	Fay	Australia	Vineyard	S	S	A
YPS606	Sanger	Pennsylvania, USA	Oak	R	R	A
YPS128	Sanger	Pennsylvania, USA	Oak	R	R	A
DBVPG1106	Sanger	Australia	Grapes	R	R	A
BC187	Sanger	Napa Valley, USA	Barrel fermentation	R	R	A
L-1374	Sanger	Chile	Wine	R	R	A
L-1528	Sanger	Chile	Wine	R	R	A
NCYC110	Sanger	West Africa	Ginger beer from *Z. officinale*	R	R	A
NCYC361	Sanger	Ireland	Beer spoilage strain from wort	R	R	A
K11	Sanger	Japan	Shochu Sake strain	R	R	A
Y9	Sanger	Japan	Ragi (similar to sake wine)	R	R	A
Y12	Sanger	Africa	Palm wine strain	R	R	A
YS2	Sanger	Australia	Baker strain	R	R	A
YS4	Sanger	Netherland	Baker strain	R	R	A
YS9	Sanger	Singapore	Baker strain	R	R	A
UWOPS83-787.3	Sanger	Bahamas	Fruit, Opuntia stricta	R	R	A
UWOPS87-2421	Sanger	Hawaii	Cladode, Opuntia megacantha	R	R	A
UWOPS03-461.4	Sanger	Malaysia	Nectar, Bertam palm	R	R	A
UWOPS05-217.3	Sanger	Malaysia	Nectar, Bertam palm	R	R	A
UWOPS05-227.2	Sanger	Malaysia	Trigona, Bertam palm	R	R	A
322134S	Sanger	RVI, Newcastle UK	Clinical isolate (Throat-sputum)	R	R	A
378604X	Sanger	RVI, Newcastle UK	Clinical isolate (Sputum)	R	R	A
DBVPG6765	Sanger	Unknown	Unknown	R	R	A
YJM978	Sanger	Bergamo, Italy	Clinical isolate (Vaginal)	R	R	A
YJM981	Sanger	Bergamo, Italy	Clinical isolate (Vaginal)	R	R	A
YJM975	Sanger	Bergamo, Italy	Clinical isolate (Vaginal)	R	R	A
DBVPG6044	Sanger	West Africa	Bili wine	R	R	A
DBVPG1788	Sanger	Finland	Soil	R	R	A
DBVPG1373	Sanger	Netherland	Soil	R	R	A
Y55	Sanger	France	Wine	R	R	A
RM11-1a	Broad	California	Wine	R	R	A
YJM789	Stanford	NA	Clinical isolate (Lung)	R	R	A

**Figure 2:**
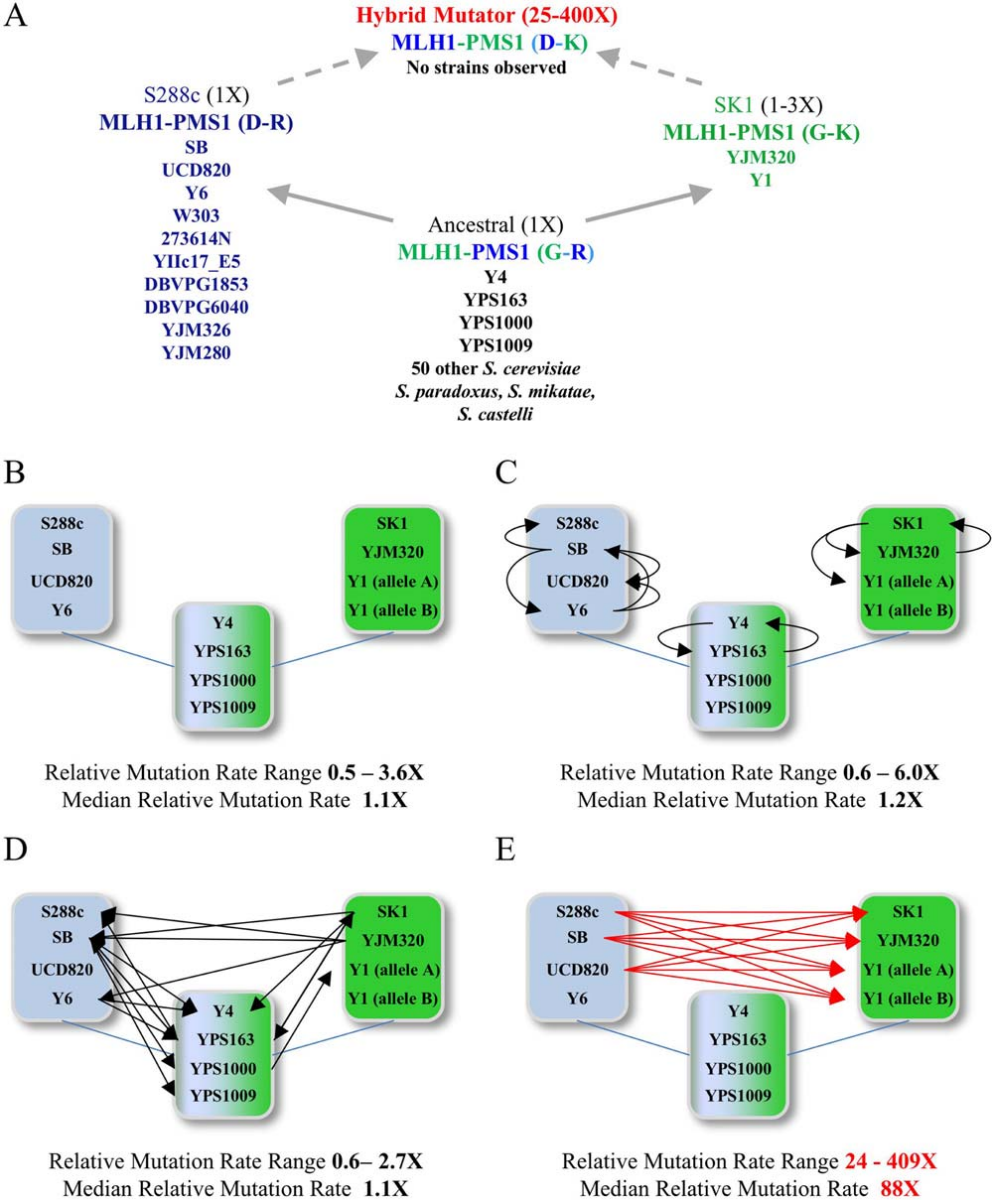
The incompatible genotype displays genetic variation that can be mapped to MLH1-PMS1. (A) 68 *S. cerevisiae* wild, clinical, production, and lab strains from world-wide collections (Table 1) are schematically grouped according to their amino acid residues 761 (G or D) in MLH1 and 818 (R or K) in PMS1. In this reconstruction, the S288c (D-R) and SK1 (G-K) strains diverged from an Ancestral (G-R) population. Genetic exchange between D-R and G-K strains would generate a mutator combination (D-K) at a 25% frequency. Arrows indicate transitions between genotypes resulting from single mutational events. The relative mutation rates of the indicated genotypes, based on Lys^+^ reversion experiments using the S288c strain (1× equals the wild-type S288c mutation rate, 2.18×10^−7^ (1.53–3.82×10^−7^, 95% C. I.) mutations per cell per generation) are shown. Additional strains were identified in each of the three compatible combinations but no strains were identified that contained the mutator incompatible combination (D-K). (B–E) For select strains from each category, *MLH1* and *PMS1* combinations (all expressed from S288c promoters) were tested using the *lys2::insE-A_14_* mutator assays in the S288c strain EAY1365. Arrows indicate combinations tested, with the tail of the arrow indicating the origin of *MLH1* used in assay and head of arrow indicating origin of *PMS1* used in assay. Panels B–D show intra-strain combinations (B, *MLH1* and *PMS1* are from the same strains), intra-group combinations (C, *MLH1* and *PMS1* are from the same group) and inter-group combinations (D, *MLH1* and *PMS1* from predicted compatible combinations). Panel E shows inter-group combinations predicted to display an incompatible phenotype.

*MLH1* and *PMS1* from the above strains were sequenced in-house (S) or were obtained from publicly available data bases (R; http://www.yeastgenome.org/; http://www.sanger.ac.uk/Teams/Team71/durbin/sgrp/; http://www.broad.mit.edu/annotation/fungi/comp_yeasts/). * For more details see: http://wiki.yeastgenome.org/index.php/CommunityW303.html. In the class column strains were grouped (see Figure 2) with respect to MLH1 761 (G or D) and PMS1 818 (R or K). The S288c-like genotype (MLH1-D, PMS1-R) is indicated as **C**, the SK1-like genotype (MLH1-G, PMS1-K) as **K**, and the predicted ancestral genotype (MLH1-G, PMS1-R) as **A**.

Of the 68 strains examined, eleven (16.2%) shared the S288c MLH1-Asp, PMS1-Arg combination, three (4.4%) shared the SK1 MLH1-Gly, PMS1-Lys combination, and the remainder 54 (79%) shared the ancestral MLH1-Gly, PMS1-Arg combination (Figures 1 and 2A, Table 1). No strains contained the MLH1-Asp, PMS1-Lys incompatible combination. One of the strains, Y1, was heterozygous for SNPs in both *MLH1* and *PMS1* but was homozygous for the MLH1-Gly, PMS1-Lys combination seen in the SK1 strain.

The neighbor joining tree analysis allowed us to examine the phylogenetic relationship between strains in each of the three categories. The eleven strains containing MLH1-Asp 761 (S288c class) are highlighted in Figure 1A. Seven of these fall into the same clade, suggesting a single, recent origin of this allele, although the presence of strains carrying MLH1-Gly 761 in this clade, and of MLH1-Asp 761 in other haplotypes, are consistent with the explanation that some recombination has occurred. The strains in this class display world-wide geographic origins, including Europe, Africa, Asia and North America (Table 1). In addition, these strains include clinical, wild, and fermenting yeasts, and thus do not share an obvious functional origin. Thus while we observed molecular evidence for a common origin of the MLH1-Asp 761 site, we were unable to conclude definitively that this allele arose in geographic isolation from the PMS1 Lys 818/822 mutation with which it is incompatible. Recent studies suggest that evidence for geographic isolation will be difficult to obtain in *S. cerevisiae* strains due to their wide usage and the ease of transfer in a globalized society [17],[20]. It is difficult to draw conclusions concerning the origin of the PMS1-Lys 818 allele, since only four such alleles were identified (Figure 1B). Finally, it is worth noting that there is a lack of concordance between the *MLH1* and *PMS1* trees with respect to individual isolates. This likely reflects the presence of outcrossing between strains in natural populations of yeast [11].

Heck et al. [11] predicted that no strains would be found that contain the MLH1-Asp, PMS1-Lys combination due to the fitness cost resulting from an elevated mutation rate. They also predicted that MLH1-Asp, PMS1-Lys combinations created in the laboratory would show a mutator phenotype regardless of allele origin. We tested the latter prediction by cloning *MLH1* and *PMS1* from a total of eleven strains into plasmids expressing *MLH1* and *PMS1* through S288c versions of their respective promoters. Four of these strains were from the S288c class (S288c, SB, UCD820, and Y6), three were from SK1 (SK1, YJM320, Y1), and four from the ancestral class (Y4, YPS163, YPS1000, YPS1009). These alleles were introduced as combinations predicted to be compatible and incompatible into the S288c strain EAY1365. The mutator phenotype of the resulting strains was measured in the highly sensitive *lys2-A14* mutator assay, which displays a roughly 10,000-fold range in mutation rate (mutations per cell per generation) between wild-type and MMR defective strains ([21]; Table 2, Figure 2B–E; Materials and Methods). All of the natural isolate combinations, in which both the *MLH1* and *PMS1* alleles originated from the same strain background, displayed mutation rates similar to those reported previously for the wild-type SK1 and S288c strains (∼1- to 3-fold relative to S288c). The one exception involved a combination involving one of the two Y1 *MLH1* alleles; this strain displayed a partial defect in MMR (Table 2). This partial defect in MMR is likely recessive and may not have an effect because Y1 appears to be an obligate diploid; we were unable to sporulate this strain under a variety of conditions.

**Figure 3:**
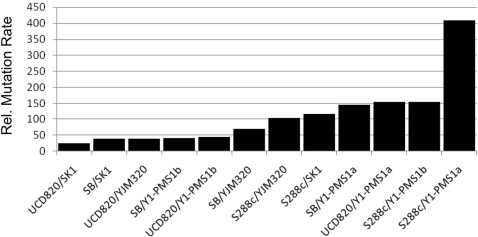
Histogram showing the incompatible combinations presented in Table 2. Mutation rates (mutations per cell per generation) are presented relative to the S288c strain (Table 2).

The median relative mutation rates for the intra-strain, intra-group, and inter-group compatible combinations were indistinguishable (1.1, 1.2, 1.1 fold above the S288c mutation rate, respectively; Figure 2). Only strains bearing the MLH1-Asp, PMS1-Lys incompatible combination displayed a mutator phenotype that was significantly different from the natural isolate class (Figure 2E compared to 2B, C, and D). The median mutation rate for the predicted incompatible class was 88, with a range from 24- to 409-fold above the S288c mutation rate. A histogram plot of the incompatible combinations suggests a continuum of mutator phenotypes (Figure 3).

**Table 2:** Median mutation rates for the indicated *MLH1-PMS1* combinations.

MLH1	PMS1	Lys^+^ reversion rate (10^−7^) (95% C. I.)	Rel Mut Rate
empty vector	S288c	14200 (8510–18000)	6514
S288c	empty vector	16000 (10540–18000)	7339
**S288c category**			
S288c	S288c	2.18 (1.53–3.82)	1.0
SB	SB	1.58 (1.31–2.30)	0.7
Y6	Y6	2.81 (2.05–3.49)	1.3
UCD820	UCD820	2.98 (2.19–3.49)	1.4
**SK1 category**			
SK1	SK1	7.92 (5.14–12.6)	3.6
YJM320	YJM320	3.61 (3.18–4.52)	1.7
Y1-MLH1a	Y1-PMS1a	6.26 (4.26–12.5)	2.9
Y1-MLH1a	Y1-PMS1b	3.77 (2.77–4.94)	1.7
Y1-MLH1b	Y1-PMS1a	9120 (5550–11200)	4184
Y1-MLH1b	Y1-PMS1b	5670 (3910–9230)	2601
**Ancestral category**			
YPS1009	YPS1009	1.03 (0.71–2.40)	0.5
YPS1000	YPS1000	1.34 (1.09–2.03)	0.6
Y4	Y4	1.28 (0.88–1.79)	0.6
YPS163	YPS163	1.78 (1.25–2.39)	0.8
**Incompatible combinations**			
UCD820	SK1	53 (28.8–53.9)	24.3
SB	SK1	84.2 (44.2–188)	38.6
UCD820	YJM320	85.8 (63.3–160)	39.4
SB	Y1-PMS1b	90.1 (71.1–134)	41.3
UCD820	Y1-PMS1b	97.8 (64.9–178)	44.9
SB	YJM320	154 (79.4–406)	70.6
S288c	YJM320	228 (144–286)	104.6
S288c	SK1	254 (184–321)	116.5
SB	Y1-PMS1a	318 (236–548)	145.9
UCD820	Y1-PMS1a	335 (299–443)	153.7
S288c	Y1-PMS1b	336 (140–508)	154.1
S288c	Y1-PMS1a	891 (748–1250)	408.7
**Compatible combinations**			
SK1	YPS163	1.27 (1.00–1.41)	0.6
YJM320	Y6	1.33 (0.92–2.29)	0.6
YPS163	Y4	1.33 (0.86–1.80)	0.6
Y4	YPS163	1.38 (0.94–1.68)	0.6
YPS163	S288c	1.59 (0.91–2.77)	0.7
SK1	SB	1.60 (1.28–2.60)	0.7
YPS163	SB	1.66 (1.13–2.46)	0.8
YJM320	S288c	1.84 (1.24–2.54)	0.8
YJM320	SB	1.91 (0.92–3.07)	0.9
SB	UCD820	2.19 (1.14–5.58)	1.0
SB	YPS163	2.26 (1.02–4.30)	1.0
SB	S288c	2.28 (1.74–3.94)	1.0
SB	Y6	3.34 (1.22–3.12)	1.1
SK1	Y4	2.39 (1.05–6.18)	1.1
SB	YPS1009	2.44 (1.36–3.51)	1.1
SB	YPS1000	2.48 (1.45–2.77)	1.1
SB	Y4	2.60 (1.75–3.61)	1.2
Y6	YPS163	2.64 (1.90–5.78)	1.2
Y6	Y4	2.70 (2.12–6.25)	1.2
Y6	SB	3.12 (2.77–3.66)	1.4
YJM320	SK1	4.15 (3.06–6.92)	1.9
Y6	UCD820	4.22 (2.34–6.01)	1.9
YPS1000	Y1-PMS1a	4.56 (3.24–8.69)	2.1
YPS163	SK1	5.87 (2.39–10.8)	2.7
SK1	YJM320	6.66 (4.64–15.8)	3.1
SK1	Y1-PMS1a	13.0 (7.50–18.5)	6.0
**Modifier Combinations**			
S288c-L271P	SK1	58.7 (45.7–78.4)	26.9
S288c-S452G	SK1	222 (145–301)	101.8
S288c-L607F	SK1	247 (178–399)	113.3

The S288c strain EAY1365 was transformed with *ARS CEN* plasmids containing *MLH1* and *PMS1* from the indicated strains (Table 1). Mutation rates (mutations per cell per generation) were determined using the *lys2::insE-A_14_* reversion assay as described in the Materials and Methods. For each strain, 15–30 independent cultures, from three to six independent transformants, were assayed. See Figure 2 for class groupings. 95% C. I. = 95% confidence interval.

### Specific Amino Acid Polymorphisms in MLH1 and PMS1 Act as Intragenic Modifiers of the Incompatible Phenotype

**Figure 4:**
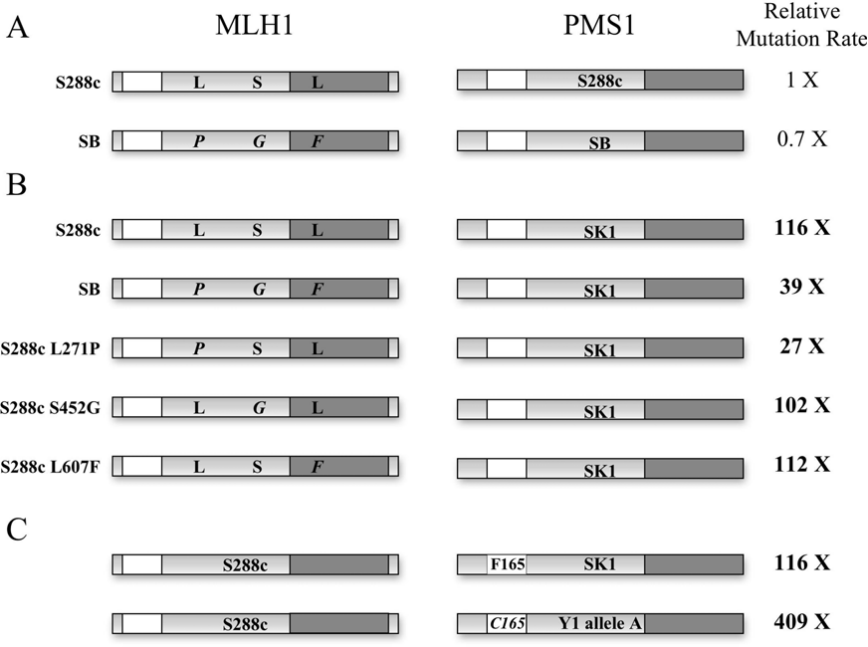
Identification of SNP modifiers of the *MLH1-PMS1* incompatibility within *MLH1* and *PMS1*. The relative mutation rate (mutations per cell per generation) in the *lys2::insE-A_14_* reversion assay was determined for each of the indicated *MLH1-PMS1* combinations (Table 2 and Materials and Methods). *MLH1-PMS1* compatible combinations (S288c, S288c; SB, SB) are shown in (A) and *MLH1-PMS1* mutator incompatible combinations (S288c, SK1; SB, SK1; S288c, Y1 Allele A) are shown in (B) and (C). Functional motifs of the two proteins are pictorially represented. The unshaded areas represent the ATP binding regions of the proteins [22], and the darker gray areas represent the MLH1-PMS1 interaction domain [52]. The amino acid variants in S288c and SB MLH1 (residues 271, 452, 607) and the site-specific mutations in S288c MLH1 (L271P, S452G, L607F) are indicated in (B) and the variants in SK1 and Y1 allele A PMS1 (residue 165) are indicated in (C).

We observed a roughly 20-fold range in mutation rate for the predicted incompatible combinations (Table 2). Because all of the *MLH1* and *PMS1* genes were expressed through S288c promoters in the same strain background, we suspected that SNPs within the open reading frames of *MLH1* and *PMS1* were responsible for modifying the incompatible phenotype. As shown previously, the S288c *MLH1*, SK1 *PMS1* combination conferred a rate that was ∼100-fold (116-fold in this study) higher than the S288c *MLH1*, S288c *PMS1* rate [11]. In contrast, S288c *MLH1*, Y1 *PMS1-allele A* and SB *MLH1*, SK1 *PMS1* combinations displayed 409- and 39-fold higher mutation rates, respectively. DNA sequence analysis allowed us to map the amino acid polymorphisms responsible for the different rates (Figure 4). SK1 *PMS1* and Y1 *PMS1-allele A* differ by only one SNP, a non-synonymous mutation causing the F165C change. Therefore this change is responsible for the difference in mutation rate. This change is located near the conserved ATP binding domain motifs within the N-terminus of MLH1 [22]. In contrast, four SNPs were found between S288c *MLH1* and SB *MLH1*, one synonymous and three non-synonymous. We tested each non-synonymous SNP in S288c *MLH1* paired with SK1 *PMS1*. In only one case, S288c *MLH1-L271P*, SK1 *PMS1*, did we see a decrease in the mutation rate (27-fold) that was similar to that seen in the SB *MLH1*, SK1 *PMS1* combination. This polymorphism is located near the putative DNA binding region of *MLH1* [23]. It is important to note that the SB *MLH1*, SB *PMS1* combination showed a mutation rate that was lower than the S288c *MLH1*, S288c *PMS1* combination. However the fact that the SB modifier only affected the incompatible combination (Figure 2, Table 2) indicates that it specifically affected the penetrance of the mutator phenotype. This work provides a unique example in which both an incompatibility and modifiers of the incompatibility have been directly mapped.

### Discussion

In Heck et al. [11] we developed a model to explain the incompatibility observed for MMR genes in crosses between the *S. cerevisiae* S288c and SK1 strains. In this model, S288c and SK1 *S. cerevisiae* strains diverged from an ancestral population, with an incompatibility occurring in 25% of the progeny obtained in crosses between the strains (Figure 2). To rigorously test this model we genotyped 68 *Saccharomyces cerevisiae* strains and found that while the vast majority belonged to the ancestral class (79.4% allele frequency), significant numbers of strains belonged to the S288c and SK1 classes (16.2 and 4.4% allele frequency, respectively). No strains were found to contain the incompatible MLH1-D761, PMS1-K818 combination that was shown previously to confer a long-term fitness cost. Consistent with the model, all MLH1-PMS1 combinations predicted to be compatible showed mutation rates similar to those seen in natural isolates. In all cases the predicted incompatible combinations displayed mutator phenotypes that were significantly higher than compatible combinations. The surprising result was that the incompatible combinations displayed mutator phenotypes that varied over a 20-fold range and could be mapped to intragenic modifiers. As described below, these results have implications for disease penetrance and the modulation of genomic mutation rates and the potential for adaptive evolution.

### Implications for Disease Penetrance

Over 500 mutations and polymorphic variations in the MMR pathway have been linked to HNPCC [24],[25]. These mutations are found in all of the major components of the pathway including *MSH2, MSH6, MLH3, MLH1*, and *PMS2* (*PMS2* is the human homolog of yeast *PMS1*) with the majority of the mutations identified in *MSH2* and *MLH1*. Many of the identified missense mutations do not show a clear link to cancer predisposition, and for roughly half of the kindreds displaying the Amsterdam criteria for HNPCC, mutation in a MMR gene has not been identified [24],[25]. In addition, many cases of colorectal cancer resembling HNPCC have been identified that display a non-Mendelian pattern of inheritance [9],[26].

Environmental and background effects have been shown to affect the penetrance of many cancers (e.g. [27],[28]). For MMR, Tanyi et al. [29] reported that the association of a non-pathogenic *hMSH2* polymorphism with an HNPCC-associated *hMSH2* mutation lowered the age of onset for the disease. Kariola et al. [9] identified individuals with colon cancer that carried two inherited polymorphisms, one in *msh2* and one in *msh6* that were inherited from separate parents. Each appeared non-pathogenic, but the inheritance of both increased cancer susceptibility. Finally, Wanat et al. [10] showed in yeast that strain background effects can alter the penetrance of HNPCC *mlh1* alleles that contain missense mutations.

In this study we examined the effects of intragenic modifiers on the mutation rate of the *MLH1-PMS1* hybrid incompatibility. We saw an ∼20-fold range in mutation rate among the different combinations of *MLH1-PMS1* that contained the hybrid D-K incompatibility (Table 2 Figures 2E, 3). This variation can be explained by specific polymorphisms within *MLH1* and *PMS1* that modify the hybrid incompatibility. We found that *MLH1-L271P* decreased the mutation rate of the S288c *MLH1*, SK1 *PMS1* combination by four-fold and *PMS1-F165C* increased the mutation rate by a similar level. However, it is clear from the continuous variation observed for the mutator phenotype (Figure 3) that additional intra- and intergenic polymorphisms are likely to modify the *MLH1-PMS1* incompatibility. While our work illustrates a relatively simple example of how background modifiers can affect disease penetrance, we believe that this will be of great interest to human geneticists who are interested in mapping modifiers that affect human disease. To our knowledge, this is the first example in which both an incompatibility and modifiers of the incompatibility have been mapped to specific amino acid variants.

### MMR and the Rapid Modulation of Genomic Mutation Rates

We were intrigued by the continuum of mutator phenotypes observed in the incompatible combination set (Figure 3). Studies in a variety of organisms and fitness studies indicated that genomic mutation rates are somewhat similar and that deviation from wild-type rates would be selected against [11],[30],[31]. In our system we observed evidence of recombination between yeast strains that would yield progeny containing the incompatible MMR combination [11]. Thus the ability to rapidly create a wide range of mutator phenotypes upon mating might be comparable to that seen in natural and laboratory populations of bacteria.

Approximately 10% of natural isolates of *E. coli* show a mutator phenotype, with about 1–3% of these strains displaying such phenotypes due to defects in the MMR pathway [32],[33]. This frequency is much higher than the rate predicted for mutators arising in a population without selection [34]. The fact that an increased mutation rate could be deleterious to the cell, but observable at high frequency within natural populations, suggests that mutators may contribute to the adaptive evolution of a population [35]–[40]. When adapting to a new environment, mutators may have an advantage due to their increased probability of acquiring the first adaptive/beneficial mutation within a population. Once adapted to an environment, however, the accumulation of deleterious mutations will outweigh the advantages of the beneficial mutation. In this model, loss or reduction of MMR functions can result in a burst of divergence, and reacquisition of fully functional MMR genes may restore fitness and sustain that divergence, owing to their role in suppressing recombination between diverged sequences. Phylogenetic studies of *E. coli* strains suggest that mutators can reacquire MMR function through horizontal gene transfer and that loss and reacquisition are selected alternately, presumably for adaptation to new environments [35].

Based on the above model and evidence for recombination between yeasts that would generate an incompatible combination, we entertain the possibility that the mating and segregation of various *MLH1* and *PMS1* alleles in yeast could result in the loss and reacquisition of MMR functions. Such events could provide a balance between fitness-loss and potential for adaptive evolution. As shown above, we were able to create a wide range of mutator phenotypes through the segregation of only two genes, indicating that a small sampling of amino acid polymorphisms could have a major effect on fitness and potentially adaptive evolution.

Could the incompatibility found between *MLH1* and *PMS1* in *S. cerevisiae* contribute to incipient speciation? Support for such an idea is based on the fact that reproductive barriers have already been shown to exist between some of the yeast strains reported in this study; for example, inter-strain crosses of SK1 and S288c show reduced spore viability of 73% [11]. We took advantage of the large databases of genome sequences obtained from yeasts isolated throughout the world to directly test MLH1-PMS1 combinations predicted to have been sampled in nature in highly sensitive mutator assays. This analysis provided molecular evidence for the S288c class of *MLH1* alleles having a single origin. However, the strains containing this allele were scattered over four continents and from many different sources including lab strains, a clinical isolate, and several domesticated strains. This is not surprising due to the ubiquity of yeast in nature, the ease of mobility of the organism, and the extensive colonization of the species for commercial use [19],[41]. It is clear from previous studies that it is difficult to assign geographic locations for even undomesticated populations of yeast. For example, in a study of wild *S. paradoxus* strains, evidence was obtained by sequence comparison for geographical divergence between North American and European strains. However, secondary contact with no admixture was recorded between these two groups from isolates found on the same tree in Pennsylvania [42]. In addition, defining species among yeasts has become difficult due to lack of pre-zygotic isolation [36],[43], lateral gene transfer [44], and introgression [20] between *Saccharomyces* species. A lack of detailed population sample data from many localities throughout the species range and a lack of understanding of the relative frequencies of clonal vs. sexual reproduction in natural populations of *S. cerevisiae* prevent a critical evaluation of the role that the MLH1-PMS1 incompatibility played in past reproductive isolation. Nonetheless, the ability to use genetic approaches in yeast to identify incompatibilities and their effects on fitness make it an attractive model to study molecular evolution.

## Materials and Methods

### Strains

*S. cerevisiae* strains (Table 1 and Figure 1) were kindly provided by Justin Fay (Washington University, St. Louis), John McCusker (Duke University), Ed Louis (University of Nottingham) and Jeff Townsend (Yale University). EAY1365 (*MATa, ura3-52, leu2Δ1, trp1Δ63, his3Δ200, lys2::insE-A14, mlh1Δ::KanMX4, pms1Δ::KanMX4*), an S288c derived strain (FY strain set, [45]), was used to measure Lys^+^ reversion rates. Yeast strains were grown in yeast extract/peptone/dextrose (YPD), minimal complete, or minimal selective media [46].

### Plasmids

The plasmids used in this study are shown in Table S3. Plasmids pEAA213 (S288c *MLH1, ARSH4 CEN6, LEU2*) and pEAA214 (SK1 *MLH1, ARSH4 CEN6, LEU2*) were described previously [2],[11]. In both vectors *MLH1* expression is driven by the S288c *MLH1* promoter. *MLH1* from different strains of yeast were cloned into pEAA213 by amplifying *MLH1* from genomic DNA [46] using Pfu turbo polymerase (Stratagene) and primers AO324 (5′ATAGTGTAGGAGGCGCTG) and AO821 (5′AACTTTGCGGCCGCGGATCCAGCCAAAACGTTTTAAAGTTA). The PCR amplified product containing the entire *MLH1* open reading frame was digested with *BamH1*-and *NheI* and inserted into the corresponding sites of pEAA213. The entire PCR fragment was DNA sequenced. All of the resulting constructs expressed *MLH1* via the S288c *MLH1* promoter.

pEAA238 (S288c *PMS1, ARSH4, CEN6, HIS3*) and pEAA239 (SK1 *PMS1, ARSH4, CEN6, HIS3*) were described previously [2],[11]. In both vectors *PMS1* expression is driven by the S288c *PMS1* promoter. *PMS1* from different strains of yeast were cloned into pEAA238 by amplifying *PMS1* from genomic DNA using Pfu turbo polymerase and primers AO548 (5′CGATTCTAATACAGATTTTAATGACC) and AO481 (5′CCACGTTCATATTCTTAATGGCTAAGC). The PCR amplified product containing the entire *PMS1* open reading frame was digested with *AatII*-and *MluI* and inserted into the corresponding sites of pEAA238. The entire PCR fragment was DNA sequenced. All of the resulting constructs expressed *PMS1* via the S288c *PMS1* promoter.

All point mutations were made in pEAA213 using the QuickChange XL Site-Directed Mutagenesis protocol (Stratagene, USA). A fragment that contained the point mutation was then subcloned into unmutagenized pEAA213 and sequenced to determine that only the desired mutations were created.

### Determination of Mutation Rates

pEAA213 (*MLH1*) and pEAA238 (*PMS1*) plasmids and derivatives were transformed into EAY1365 using standard methods [47] and were maintained on minimal histidine, leucine dropout plates. All transformants were assayed for their ability to grow on lactate as a carbon source. Because EAY1365 is a mutator strain, we were concerned that variation in genetic background between different strain isolates could affect mutation rate. For each *MLH1, PMS1* combination, 15–30 independent cultures, from three to six independent transformants derived from the same EAY1365 frozen stock, were assayed to determine the mutation rate. We found that independent transformants containing the same plasmid combination displayed mutation rates that did not deviate significantly from each other; this can be seen in the 95% confidence intervals presented in Table 2. Reversion of the *lys2::insE-A14* allele to Lys^+^ was measured as described previously [10],[11]. The reversion rate μ (mutations per cell per generation) was calculated using the equation μ = f/ln(Nμ), where f is the frequency of revertants and N is the total number of revertants in 1 ml of overnight culture ([21],[48], Dmitry Gordenin, personal communication). This equation, derived by Drake [50], is transcendental and can be solved only by iteration; we use a computer method as suggested by Dmitry Gordenin. This equation is used to calculate the mutation rate for each culture and the median of the independent cultures is defined as the mutation rate. The 95% confidence intervals were determined as described [49].

### Sequencing, Alignment, and Phylogenetic Reconstruction

The sequence of *MLH1* and *PMS1* from each of the strains in Table 1 was determined in house or taken from publicly available sequence (http://www.yeastgenome.org/; http://www.sanger.ac.uk/Teams/Team71/durbin/sgrp/; http://www.broad.mit.edu/annotation/fungi/comp_yeasts/). For the in house sequencing, *MLH1* and *PMS1* coding regions and approximately 200 bp of downstream sequence were amplified by PCR, purified and then sequenced using Big Dye chemistry and an ABI 3730xl DNA Analyzer at the Cornell Life Sciences Core Laboratories Center.

Sequences were aligned using the CLUSTALW algorithm [50], using the resources of the Computational Biology Service Unit from Cornell University, which is partially funded by Microsoft Corporation. Neighbor-Joining trees were constructed based on nucleotide sequences, using PAUP*4.0b10 [51]. For *MLH1*, the nucleotide site responsible for the D761G polymorphism was excluded from phylogenetic reconstruction; similarly, the site responsible for R818K in *PMS1* was excluded.

## Supporting Information

Table S1 *MLH1* polymorphisms.
(0.03 MB DOC)

Table S2 *PMS1* polymorphisms.
(0.04 MB DOC)

Table S3 Plasmids used in this study.
(0.02 MB DOC)

## References

[1] Brem RB, Yvert G, Clinton R, Kruglyak L (2002). Genetic dissection of transcriptional regulation in budding yeast.. Science.

[2] Argueso JL, Kijas AW, Sarin S, Heck JA, Waase M (2003). Systematic mutagenesis of the *Saccharomyces cerevisiae* MLH1 gene reveals distinct roles for Mlh1p in meiotic crossing over and in vegetative and meiotic mismatch repair.. Mol Cell Biol.

[3] Lipkin SM, Rozek LS, Rennert G, Yang W, Chen PC (2004). The MLH1 D132H variant is associated with susceptibility to sporadic colorectal cancer.. Nat Genet.

[4] Sinha H, Nicholson BP, Steinmetz LM, McCusker JH (2006). Complex genetic interactions in a quantitative trait locus.. PLoS Genet.

[5] Steinmetz LM, Sinha H, Richards DR, Spiegelman JI, Oefner PJ (2002). Dissecting the architecture of a quantitative trait locus in yeast.. Nature.

[6] Hunter N, Chambers SR, Louis EJ, Borts RH (1996). The mismatch repair system contributes to meiotic sterility in an interspecific yeast hybrid.. EMBO J.

[7] Rayssiguier C, Thaler DS, Radman M (1989). The barrier to recombination between *Escherichia coli* and *Salmonella typhimurium* is disrupted in mismatch-repair mutants.. Nature.

[8] Kunkel TA, Erie DA (2005). DNA mismatch repair.. Annu Rev Biochem.

[9] Kariola R, Otway R, Lonnqvist KE, Raevaara TE, Macrae F (2003). Two mismatch repair gene mutations found in a colon cancer patient–which one is pathogenic?. Hum Genet.

[10] Wanat JJ, Singh N, Alani E (2007). The effect of genetic background on the function of *Saccharomyces cerevisiae mlh1* alleles that correspond to HNPCC missense mutations.. Hum Mol Genet.

[11] Heck JA, Argueso JL, Gemici Z, Reeves RG, Bernard A (2006). Negative epistasis between natural variants of the *Saccharomyces cerevisiae MLH1* and *PMS1* genes results in a defect in mismatch repair.. Proc Natl Acad Sci USA.

[12] Brideau NJ, Flores HA, Wang J, Maheshwari S, Wang X (2006). Two Dobzhansky-Muller genes interact to cause hybrid lethality in *Drosophila.*. Science.

[13] Rawson PD, Burton RS (2002). Functional coadaptation between cytochrome c and cytochrome c oxidase within allopatric populations of a marine copepod.. Proc Natl Acad Sci USA.

[14] Presgraves DC, Balagopalan L, Abmayr SM, Orr HA (2003). Adaptive evolution drives divergence of a hybrid inviability gene between two species of *Drosophila*.. Nature.

[15] Wittbrodt J, Adam D, Malitschek B, Mäueler W, Raulf F (1989). Novel putative receptor tyrosine kinase encoded by the melanoma-inducing Tu locus in *Xiphophorus*.. Nature.

[16] Ting CT, Tsaur SC, Wu ML, Wu CI (1998). A rapidly evolving homeobox at the site of a hybrid sterility gene.. Science.

[17] Fay JC, McCullough HL, Sniegowski PD, Eisen MB (2004). Population genetic variation in gene expression is associated with phenotypic variation in *Saccharomyces cerevisiae*.. Genome Biol.

[18] Wei W, McCusker JH, Hyman RW, Jones T, Ning Y (2007). Genome sequencing and comparative analysis of *Saccharomyces cerevisiae* strain YJM789.. Proc Natl Acad Sci USA.

[19] Fay JC, Benavides JA (2005). Evidence for domesticated and wild populations of *Saccharomyces cerevisiae*.. PLoS Genet.

[20] Liti G, Barton DB, Louis EJ (2006). Sequence diversity, reproductive isolation and species concepts in *Saccharomyces*.. Genetics.

[21] Tran HT, Keen JD, Kricker M, Resnick MA, Gordenin DA (1997). Hypermutability of homonucleotide runs in mismatch repair and DNA polymerase proofreading yeast mutants.. Mol Cell Biol.

[22] Ban C, Yang W (1998). Crystal structure and ATPase activity of MutL: implications for DNA repair and mutagenesis.. Cell.

[23] Ban C, Junop M, Yang W (1999). Transformation of MutL by ATP binding and hydrolysis: a switch in DNA mismatch repair.. Cell.

[24] Lynch HT, de la Chapelle A (2003). Hereditary colorectal cancer.. N Engl J Med.

[25] Chung DC, Rustgi AK (2003). The hereditary nonpolyposis colorectal cancer syndrome: genetics and clinical implications.. Ann Intern Med.

[26] Viel A, Genuardi M, Capozzi E, Lenonardi F, Bellacosa A (1997). Characterization of MSH2 and MLH1 mutations in Italian families with hereditary nonpolyposis colorectal cancer.. Genes Chromosomes Cancer.

[27] Fijneman RJ (2005). Genetic predisposition to sporadic cancer: how to handle major effects of minor genes?. Cell Oncol.

[28] Gillanders EM, Xu J, Chang BL, Lange EM, Wiklund F (2004). Combined genome-wide scan for prostate cancer susceptibility genes.. J Natl Cancer Inst.

[29] Tanyi M, Olasz J, Lukacs G, Csuka O, Toth L (2006). Pedigree and genetic analysis of a novel mutation carrier patient suffering from hereditary nonpolyposis colorectal cancer.. World J Gastroenterol.

[30] Zeyl C, DeVisser JA (2001). Estimates of the rate and distribution of fitness effects of spontaneous mutation in *Saccharomyces cerevisiae*.. Genetics.

[31] Drake JW, Charlesworth B, Charlesworth D, Crow JF (1998). Rates of Spontaneous Mutation.. Genetics.

[32] LeClerc JE, Li B, Payne WL, Cebula TA (1996). High mutation frequencies among *Escherichia coli* and *Salmonella* pathogens.. Science.

[33] Matic I, Radman M, Taddei F, Picard B, Doit C (1997). Highly variable mutation rates in commensal and pathogenic *Escherichia coli*.. Science.

[34] Boe L, Danielsen M, Knudsen S, Petersen JB, Maymann J (2000). The frequency of mutators in populations of *Escherichia coli*.. Mutat Res.

[35] Denamur E, Lecointre G, Darlu P, Tenaillon O (2000). Evolutionary implications of the frequent horizontal transfer of mismatch repair genes.. Cell.

[36] Sniegowski PD, Dombrowski PG, Fingerman E (2002). *Saccharomyces cerevisiae* and *Saccharomyces paradoxus* coexist in a natural woodland site in North America and display different levels of reproductive isolation from European conspecifics.. FEMS Yeast Res.

[37] Taddei F, Radman M, Maynard-Smith J, Toupance B, Gouyon PH (1997). Role of mutator alleles in adaptive evolution.. Nature.

[38] Tanaka MM, Bergstrom CT, Levin BR (2003). The evolution of mutator genes in bacterial populations: the roles of environmental change and timing.. Genetics.

[39] Townsend JP, Nielsen KM, Fisher DS, Hartl DL (2003). Horizontal acquisition of divergent chromosomal DNA in bacteria: effects of mutator phenotypes.. Genetics.

[40] Giraud A, Matic I, Tenaillon O, Clara A, Radman M (2001). Costs and benefits of high mutation rates: adaptive evolution of bacteria in the mouse gut.. Science.

[41] Greig D (2007). Population biology: wild origins of a model yeast.. Curr Biol.

[42] Kuehne HA, Murphy HA, Francis CA, Sniegowski PD (2007). Allopatric divergence, secondary contact, and genetic isolation in wild yeast populations.. Curr Biol.

[43] de Barros Lopes M, Bellon JR, Shirley NJ, Ganter PF (2002). Evidence for multiple interspecific hybridization in *Saccharomyces* sensu stricto species.. FEMS Yeast Res.

[44] Liti G, Peruffo A, James SA, Roberts IN, Louis EJ (2005). Inferences of evolutionary relationships from a population survey of LTR-retrotransposons and telomeric-associated sequences in the *Saccharomyces* sensu stricto complex.. Yeast.

[45] Winston F, Dollard C, Riscupero-Hovasse SL (1995). Construction of a set of convenient *Saccharomyces cerevisiae* strains that are isogenic to S288C.. Yeast.

[46] Rose MD, Winston FM, Hieter P (1990). Methods in yeast genetics: a laboratory course manual.

[47] Gietz RD, Schiestl RH (2007). Quick and easy yeast transformation using the LiAc/SS carrier DNA/PEG method.. Nat Protoc.

[48] Drake JW (1991). A constant rate of spontaneous mutation in DNA-based microbes.. Proc Natl Acad Sci USA.

[49] Dixon WJ, Massey FJ (1969). Introduction to Statistical Analysis.

[50] Larkin MA, Blackshields G, Brown NP, Chenna R, McGettigan PA (2007). Clustal W and Clustal X version 2.0.. Bioinformatics.

[51] Swofford D (2002). PAUP*. Phylogenetic Analysis Using Parsimony (*and other methods).

[52] Pang Q, Prolla TA, Liskay RM (1997). Functional domains of the *Saccharomyces cerevisiae* Mlh1p and Pms1p DNA mismatch repair proteins and their relevance to human hereditary nonpolyposis colorectal cancer-associated mutations.. Mol Cell Biol.

[53] Rozas J, Sánchez-DelBarrio JC, Messeguer X, Rozas R (2003). DnaSP, DNA polymorphism analyses by the coalescent and other methods.. Bioinformatics.

