# Supplementary material for: Incompatibilities Involving Yeast Mismatch Repair Genes: A Role for Genetic Modifiers and Implications for Disease Penetrance and Variation in Genomic Mutation Rates
Source: PLoS Genet. 2008 Jun 20;4(6):e1000103. doi: 10.1371/journal.pgen.1000103 (PMC2413424; doi:10.1371/journal.pgen.1000103)
Supplement: Table S1 — MLH1 polymorphisms. (0.03 MB DOC) [file pgen.1000103.s001.doc]

**Table S1. *MLH1* polymorphisms**

10 20 30 40 50 60 70

* * * * * * *

UCD612 AAGTCAAAACCCCACTACCCGCGGCAAGCAATACAGACAAGGTTTAACATACGCAGCTAACAGCCGCCTAAC

K11 ................................G..........A......................A.....

YPS163 .........T........T.............G......G...........T........T..........T

Y1-B ..T....T.T........T.............G..................T....----------------

YPS1000 .........T........T.............G..................T........T...........

DBVPG6044 .........TT...A...T....A........G..................T........T...........

SK1 .........TT...A...T....A........G..................T........T...........

YPS1009 ........GT......G.T.A...........G..................T.....C..T...........

YJM320 .........T........T.............G..................T........T...........

UWOPS05-227.2 .........T........T.......G.....GT.................T............--------

YPS128 .........T..............T.......GT.................T........T..........T

YPS606 .........T..............T.......GT.................T........T..........T

Y1-A .....T...T.T............T.......GTG...........CT..G.....----------------

M5-7 .........T.T............T.......GTG...........CT..G.....----------------

UWOPS83-787.3 .........T.T............T.......GTG...........CT..G.....................

Y12 .........T.T............T.......GTG...........CT..G..................C..

M2-8 .........T.T.........T..T.......GTG...........CT..G.....----------------

M1-2-B .........T.T............T.......GTG...........CT..G.....----------------

M7-8 .........T.T............T.......GTG...........CT..G.....----------------

YJM627 .........T.T............T.......GTG...........CT..G.....----------------

YJM978 .........T.T............T.......GTG...........CT..G..............AG.....

UCD175 .........T.T............T.......GTG...........CT..G..............AG.....

UCD781 .........T.T............T.......GTG...........CT..G..............AG.....

L-1374 .........T.T............T.......GTG...........CT..G..............AG.....

YJM981 .........T.T............T.......GTG...........CT..G..............AG.....

RM11-1a .........T.T............T.......GTG...........CTT.G..............AG.....

YS4 T..G.....A.T............T...GTGCG.G...........CT..G..............AG.....

YS9 .........T.T............T.......GTG...........CT..G..............AG.....

UCD2120 .........T.T............T.......GTG...........CT..G..............AG.....

DBVPG1373 .........T.T............T.......GTG...........CT..G..............AG.....

YJM975 .........T.T............T.......GTG...........CT.AG..............AG.....

YS2 .........T.T............TG......GTG...........CT..G..............AG.....

DBVPG1106 .........T.T............T.......GTG...........CT..G..............AG.....

DBVPG6765 .........T.T............T.......GTG...........CT..G..............AG.....

Y55 .........T.T............T.......GTG...........CT..G..............AG.....

L-1528 .........T.T............T.......GTG.....A....GCT..G..............AG.....

UWOPS87-2421 .........T.T............T.......GTG.?...A....GCT..G..........?.A.AG.....

UCD51 .........T.T............T.......GTG.....A....GCT..G..............AG.....

UCD529 .........T.T............T.......GTG...........CT..G..............AG.....

DBVPG1788 .........T.T............T.......GTG...........CT..G..............AG.....

Y4 .........T.T............T.......GTG...........CT..G..............AG.....

Y8 .........T.T............T.......GTG...........CT..G..............AG.....

I14 .........T.T............T.......GTG...........CT..G..............AG.....

UCD765 .........T.T............T.......GTG............T..G..............AG.....

YJM269 .........T.T............T.......GTG............T..G.....----------------

B2 .........T.T............T.......GTG............T..G..............AG.....

BC187 .........T.T............T.......GT..?.........CT..G..............AG.....

M1-2-A .........T.T..........T.T.......GT.A...........T..G.....----------------

UWOPS03-461.4 T........T.T............T.......GT............CT..-T.T......T...........

Y9 .........T.T............T.......GT.............T..GTT.......T......T....

YJM789 .........T.......T......T.......GT.............T..G.........T...........

322134S .G.......T.T.......T....T-......GT.............T.AG..............AG.....

NCYC361 .........T..............T.......GT.............T..G..............AG.....

Y3 .........T..TG..........T.......GT.............T..G.....T........AG.....

NCYC110 .........T..............T.......GT..C......?.......T...........AGAG.G.G.

YJM145 .........T.......T......T.......GT.............T..G.....----------------

UWOPS05-217.3 .........T........T.............GT.............T.AG.......CG.....AG.....

378604X ....A....T........T.............G........CC.A..T..................G.....

YJM339 .........T.....A...........A....G....T.............T....----------------

273614N .........T.T............T.......GTG...........CT..G....A..C....AGAG.....

Sb .........T.T............T.......GTG...........CT..G....A.......A.AG.....

W303 .........T.......T......T....T..GT.............T..G...CA.........AT...G.

S288c .........T.......T......T.......GT.............T..G....A.......AGAG.G.G.

Y6 .........T.......T......T.......GT.............T..G....A......AA.AG.?.?.

UCD820 ......G..T..............T.......GT.............T..G....A.......A.AG.....

YJM326 .........T.....A........T.......GT.............T..G....A.........AG.....

DBVPG1853 .........T.......T......T.......GT....G........T..G....A....A....AG.....

DBVPG6040 .........T.......T......T.......GT.............T..G....A.....T...AG.....

YIIc17_E5 .........T.T............T.......GT.A...........T..G....A......AA.AG.....

YJM280 .........T.T............T.......GT.A...........T..G....A.........AG.....

[1] nt_1064 [2] nt_1160 [3] nt_1227 [4] nt_1262 [5] nt_1278

[6] nt_1291 [7] nt_1315 [8] nt_1333 [9] nt_1361 [10] nt_1421

[11] nt_1487 [12] nt_1553 [13] nt_1570 [14] nt_1619 [15] nt_1721

[16] nt_1727 [17] nt_1741 [18] nt_1813 [19] nt_1835 [20] nt_1844

[21] nt_1904 [22] nt_1927 [23] nt_1964 [24] nt_1998 [25] nt_2046

[26] nt_2057 [27] nt_2102 [28] nt_2139 [29] nt_2159 [30] nt_2160

[31] nt_2174 [32] nt_2182 [33] nt_2225 [34] nt_2264 [35] nt_2408

[36] nt_2452 [37] nt_2467 [38] nt_2468 [39] nt_2528 [40] nt_2536

[41] nt_2582 [42] nt_2588 [43] nt_2589 [44] nt_2590 [45] nt_2593

[46] nt_2848 [47] nt_2885 [48] nt_2940 [49] nt_2968 [50] nt_2969

[51] nt_3101 [52] nt_3177 [53] nt_3295 [54] nt_3299 [55] nt_3321

[56] nt_3352 [57] nt_3382 [58] nt_3435 [59] nt_3713 [60] nt_3716

[61] nt_3722 [62] nt_3725 [63] nt_3752 [64] nt_3850 [65] nt_3923

[66] nt_3945 [67] nt_3972 [68] nt_4131 [69] nt_4140 [70] nt_4191

[71] nt_4369 [72] nt_4396

_________________________________________________________________________________________

Haplotype structure surrounding the incompatible single nucleotide polymorphisms in *MLH1* for all strains of *S. cerevisiae* was determined using the computer program DnaSP [53]. The nucleotide change underlying the MLH1-D761 polymorphism is highlighted in blue, and the coding region spans nucleotides 1001-3307 (including polymorphic sites 1-54). “.” indicates a match to the reference sequence (UCD612), and “-“ indicates missing data. Locations of each polymorphic site in the full sequence alignment are indicated below.
