# Supplementary material for: Incompatibilities Involving Yeast Mismatch Repair Genes: A Role for Genetic Modifiers and Implications for Disease Penetrance and Variation in Genomic Mutation Rates
Source: PLoS Genet. 2008 Jun 20;4(6):e1000103. doi: 10.1371/journal.pgen.1000103 (PMC2413424; doi:10.1371/journal.pgen.1000103)
Supplement: Table S2 — PMS1 polymorphisms. (0.04 MB DOC) [file pgen.1000103.s002.doc]

**Table S2. *PMS1* polymorphisms**

10 20 30 40 50 60 70 80 90 100 110 120 130

* * * * * * * * * * * * *

YPS1009 CAGAGTGTAGCAGAGCAGAGTATATCCACTGTAGTCACCGTATTTATTCGCCCTGTGTCCGAGCAGATCGCGCTGC-AGAAGTACGTTCGCTAATTCGAAGCTTATGAAGTGGGCCATTTTCGG----------

YPS163 ............................................................................-...............................................----------

UCD612 .?...........................C...............G......................T..TT..T-.......T....C....C....GA...G...G.GA............----------

273614N ..A...........A..........G.GGCA...C..GT....C.G..TAT..........T...T...C.....T-.......T....C.......A..A...G...................----------

DBVPG1853 ..A...........A..........G.GGCA...C..GT....C.G..TAT..........T...T.C.C....TT-.......T....C.......A..A...G...................----------

DBVPG6040 T.A...........A............GGCA...CT.GT....C.G..TAT..........T...T...C.....T-.......T....C.......A..AT..G...................----------

YIIc17_E5 .............................C...............G......................T..TT..T-.......T....C....C.....A...G...G.GA............----------

Sb ..A...........A..........G.GGCA...C..GT....C.G..TAT..........T...T.C.C....TT-.......T....C.......A..A...G...................----------

I14 ..A...........A............GGCAC......T......G...A..T.C.A..................T-.......T....C....C.....A...G.....GA............----------

UCD2120 ..A...........A..........G.GGCA...C..GT....C.G..TAT..........T...T...C.....T-.......T....C.......A..A...G...................----------

Y8 ..A...........A..........G.GGCA...C..GT....C.G..TAT..........T...T...C.....T-.......T....C.......A..A...G............-----------------

B2 .CA...........A..........G.GGCA...C..GT....C.G..TAT..........T...T...C....?T-.......T....C.......A..A...G............-----------------

Y4 .CA...........A..........G.GGCA...C..GT....C.G..TAT..........T...T...C.....T-.......T....C.......A..A...G............-----------------

UCD529 ..A...........A..........G.GGCA...C..GT....C.G..TAT..........T...T...C.....T-.......T....C.......A..A...G...................----------

UCD175 ..A...........A..........G.GGCA...C..GT....C.G..TAT..........T...T...C.....T-.......T....C.......A..A...G...................----------

Y6 ..A...........A............GGCA...C..GT.C..C.G..TAT..........T...T.........T-G..C...T....C....C.....A...G.A...GA............----------

UCD820 -.A...........A..........G.GGCA...C..GT....C.G..TAT..........TC..T...C.....T-.....A.T....C.....G.A..A...G...................----------

322134Sn -----------------------------CA...C..GT....C.G...A.........................T-.......T....C.......A..AT..G...................TGGGCTATGA

UWOPS05-217.3 -----------------------------CA...C..GT....C.G...A.........................T-.......T....C.......A..A...G...................TGGGCCATGA

DBVPG6044 -----------------------------CA..............G.C....................T..T...T-....A..T...AC.C.G..A...A...G...................TGGGCCACGA

NCYC110 -----------------------------CA.....?........G......................T..T..CT-.......T....C..........A...G...................GGGGCCACGA

YPS128 -----------------------------.......................................T..T...T-....A..T...AC..................................TGGACCATAA

YPS606 -----------------------------................G..............................-.......................?.......................TGGACCATAA

K11 -----------------------------C...............G.......?..A...........T..TT..T-.......T....C....C.....A...G...G.GA..T.........TGGGCCATGA

Y12 -----------------------------C...............G......................T..TT..T-.......T....C....C.....A...G...G.GA.A..........TGAGCCATGA

UWOPS83-787.3 -----------------------------C...............G......................T..TT..T-.......T.AG.C....C.....A...G.....GA...A........TGGGCCATGA

Y9 -----------------------------C...............G......................T..TT..T-.......T....C....C.....A.G.G.....GA............TGGGCCATGA

YJM339 -----------------------------CA.......T......G...A.....CA......T....T....C.T-.A.....T....C....C.....A...G.....GA............TGGGCCATAA

YPS1000 -----------------------------CAC......T......G...A..T.C.A............C.....T-.......T....C....C.....A...G.....GA.....-----------------

YJM326 -----------------------------CA...C..GT....C.G..T...................T..TT..T-.......T....C....C...T.A..CG.....GA............TTGGTCACGA

UWOPS87-242.1 -----------------------------CA..............G......................TCGTT..T-.......T....C....C.....A...G...G.GA.A..........TGAGCTCTGA

YS9 -----------------------------CA..............GA....T..C.A...........T..TT..T-.......T....C.......A..A...G...................TGGGCTCTGA

UWOPS03-461.4 -----------------------------CA.G.....T......G...A......A.T....T...........TT.......T....C..G.......A...GCA...GA....G......TTGGGCCATGA

UWOPS05-227.2 -----------------------------CA.......T......G...A......A.T....T.-.........TC..C....T....C..G.......A...GCA...GA....G.CCAG..TGGGCCATGA

Y3 -----------------------------CA.......T......G...A.....C.......TC...TC.....T-.......T....C.......A..A...G............-----------------

BC187 -----------------------------CA...C..GT....C.G..TA.........................T-.......T....C.......A..A...G...................TGGGCCATGA

NCYC361 -----------------------------CA...C.TGT....C.G..TA.........................T-.......T....C.......A..A...G.........A.T.......TGGGCCATGA

DBVPG110 -----------------------------CA...C..GT....C.G..TA.........................T-.......T....C.......A..A...G...................TGGGCCATGA

DBVPG6765 -----------------------------CA...C..GT....C.G..TAT..............T...C.....T-..G....T....C.......A..A...G...................TGGGCCATGA

YJM975 -----------------------------CA......GT....C.G..TAT..........C...T...C.....T-.......T....C.......A..A...G...................TGGGCCATGA

YS2 -----------------------------CA...C..GT....C.G..TAT..........G...T...C.....T-.......T....C.......A..A...G...................TGGGCCATGA

378604X -----------------------------CA...C..GT....C.G..TAT..........T...T...C.....T-.......T....C.......A..A...G...................GGGGCCATGA

YJM981 -----------------------------CA...C..GT...AC.G..AAT..A.......T...T...C.....T-.......T....C.......A..A...G....C..............TGGGCCATGA

YJM280 -----------------------------CA...C..GT....C.G..TAT........TTT...T...C.....T-.......T....C.......A..A...G...................TGGGCCATGA

L-1528 -----------------------------CA...C..GT....C.G..TAT..........T...T...C.....T-.......T....C.......A..A...G...................TGGGCCATGA

YJM627 -----------------------------CA...C..GT....C.G..TAT..........T...T...C.....T-.......T....C.......A..A...G...................TGGGCCATGA

L-1374 -----------------------------CA...C..GT....C.G..TAT..........T...T...C.....T-.......T....C.......A..A...G...................TGGGCCATGA

Y55 -----------------------------CA...C..GT....C.G..TAT..........T...T...C.....T-.......T....C.......A..A...G...................TGGGCCATGA

DBVPG1373 -----------------------------CA...CT.GT....C.G..TAT..........T...T...C.....T-.......T....C.......A..A...G...................TGGGCCATGA

S288C T.A...........A............GGCA...CT.GT....C.G..TAT..........T...T...C.....T-.......T....C.......A..A...G...................TGGGCCATGA

YJM978 -----------------------------CA...C..GTA...C.G..TAT.......A..T...T...C.....T-.......T....C.......-..A...G...................TGGGCTCTGC

YS4 -----------------------------CA...CT.GT....C.G..TAT..........T...T...C.....T-.......T....C.......A..A...G...................TGGGCTCTGG

W303 -----------------------------CA...CT.GT....C.G..TAT..........T...T...C.....T-.......T....C.......A..AT..G...................TGGGCCATGG

YJM789 -----------------------------CA...C..GT....C.G..TAT..........T...T...C.....T-.......T....C.......A..A...G............-----------------

UCD781 -----------------------------CA...C..GT....C.G..TAT..........T...T...C.....T-.......T....C.......A..A...G............-----------------

YJM269 -----------------------------CA...C..GT....C.G..TAT..........T...T...C.....T-.......T....C.......A..A...G...................TGGGCCATGA

DBVPG1788 -----------------------------CA...C..GT....C.G..TAT..........T...T...C.....T-.......T....C.......A..A...G..T..............A.TGGGCCATGA

UCD765 -----------------------------CA...C..GT....C.G..TAT..........T...T...C.....T-.......T....C.......A..A...G............-----------------

RM11-1a -----------------------------CA...C..GT....C.G..TAT..........T...T...C.....T-.......T....C.......A..A...G............-----------------

M7-8 -----------------------------CA...C..GT....C.G..TAT..........T...T...C.....T-.......T....C.......A..A...G............-----------------

M5-7 -----------------------------CA...C..GT....C.G..TAT..........T...T...C.....T-.......T....C.......A..A...G............-----------------

M2-8 -----------------------------CA...C..GT....C.G..TAT..........T...T...C.....T-.......T....C.......A..A...G............-----------------

M1-2-B -----------------------------CA...C..GT..T.C.G..TAT..........T...T...C....TT-.......T....C.......A..A...G............-----------------

M1-2-A -----------------------------CA...CT.GT....C.G..TAT..........T...T...C.....T-.......T....C.......A..AT..G............-----------------

UCD51 ---GAGTGTAGCAGAACAGAGTATATGGGCA...C..GT....C.G..TAT..........T...T...C.....T-.......T....C.......A..A...G............C......----------

SK1 -----------------------------C...............GA.......C.A..................T-......TTC...C....C.....A...G.....GAA.........A.TGGGCCATGA

YMJ320 -----------------------------CAC.A....T......G...........C........T.T..TT..T-.......T....CT...C.....A...G...G.GAA.........C.TGGGCCATGA

Y1-A .............................C..............GGA.......C.A..................T-......TTC...C....C.....A...G.....GAA...........TGGG------

Y1-B -----------------------------CA............C.GA.TAT..........T...T...C.A...T-.......T....C.......A..A...G.....GAA....-----------------

[1] nt_919 [2] nt_921 [3] nt_937 [4] nt_951 [5] nt_957

[6] nt_959 [7] nt_960 [8] nt_961 [9] nt_962 [10] nt_964

[11] nt_965 [12] nt_966 [13] nt_970 [14] nt_972 [15] nt_973

[16] nt_974 [17] nt_975 [18] nt_976 [19] nt_977 [20] nt_978

[21] nt_980 [22] nt_981 [23] nt_982 [24] nt_983 [25] nt_984

[26] nt_985 [27] nt_986 [28] nt_991 [29] nt_1005 [30] nt_1125

[31] nt_1130 [32] nt_1185 [33] nt_1198 [34] nt_1218 [35] nt_1221

[36] nt_1266 [37] nt_1277 [38] nt_1346 [39] nt_1348 [40] nt_1373

[41] nt_1406 [42] nt_1426 [43] nt_1449 [44] nt_1482 [45] nt_1511

[46] nt_1521 [47] nt_1569 [48] nt_1574 [49] nt_1575 [50] nt_1728

[51] nt_1731 [52] nt_1734 [53] nt_1737 [54] nt_1781 [55] nt_1830

[56] nt_1839 [57] nt_1875 [58] nt_1878 [59] nt_1886 [60] nt_1940

[61] nt_1947 [62] nt_1961 [63] nt_1990 [64] nt_2041 [65] nt_2152

[66] nt_2189 [67] nt_2214 [68] nt_2216 [69] nt_2230 [70] nt_2238

[71] nt_2239 [72] nt_2240 [73] nt_2382 [74] nt_2423 [75] nt_2430

[76] nt_2448 [77] nt_2450 [78] nt_2477 [79] nt_2490 [80] nt_2497

[81] nt_2500 [82] nt_2569 [83] nt_2615 [84] nt_2617 [85] nt_2630

[86] nt_2660 [87] nt_2678 [88] nt_2681 [89] nt_2733 [90] nt_2754

[91] nt_2778 [92] nt_2817 [93] nt_2827 [94] nt_2838 [95] nt_2872

[96] nt_2876 [97] nt_2902 [98] nt_2913 [99] nt_2948 [100] nt_3089

[101] nt_3149 [102] nt_3170 [103] nt_3175 [104] nt_3191 [105] nt_3261

[106] nt_3275 [107] nt_3338 [108] nt_3364 [109] nt_3418 [110] nt_3419

[111] nt_3438 [112] nt_3480 [113] nt_3569 [114] nt_3597 [115] nt_3615

[116] nt_3729 [117] nt_3733 [118] nt_3764 [119] nt_3781 [120] nt_3782

[121] nt_3784 [122] nt_3788 [123] nt_3822 [124] nt_3832 [125] nt_3909

[126] nt_3927 [127] nt_3940 [128] nt_3952 [129] nt_4095 [130] nt_4151

[131] nt_4152 [132] nt_4206 [133] nt_4230 [134] nt_4232

Haplotype structure surrounding the incompatible single nucleotide polymorphisms in *PMS1* for all strains of *S. cerevisiae* was determined using the computer program DnaSP [53]. The nucleotide change underlying the PMS1-R822 polymorphisms is highlighted in green, and the coding region spans nucleotides 1007- 2629 (including polymorphic sites 30-84). “.” indicates a match to the reference sequence (YPS1009), and “-“ indicates missing data. Locations of each polymorphic site in the full sequence alignment are provided below.
