# Supplementary material for: Incompatibilities Involving Yeast Mismatch Repair Genes: A Role for Genetic Modifiers and Implications for Disease Penetrance and Variation in Genomic Mutation Rates
Source: PLoS Genet. 2008 Jun 20;4(6):e1000103. doi: 10.1371/journal.pgen.1000103 (PMC2413424; doi:10.1371/journal.pgen.1000103)
Supplement: Table S3 — Plasmids used in this study. (0.02 MB DOC) [file pgen.1000103.s003.doc]

**Table S3. Plasmids used in this study**

Strain MLH1 PMS1

­­­­­­­­­_____________________________________________________________________

S288c pEAA213 pEAA238

S288c chimera pEAA419 (S288c MLH1-L271P)

S288c chimera pEAA420 (S288c MLH1-S452G)

S288c chimera pEAA421 (S288c MLH1-L607F)

SK1 pEAA214 pEAA239

YJM320 pEAA352 pEAA353

Y1 allele A pEAA354 pEAA356

Y1 allele B pEAA355 pEAA357

UCD820 pEAA358 pEAA359

YPS1000 pEAA360 pEAA361

Y6 pEAA362 pEAA363

SB pEAA364 pEAA365

YPS1009 pEAA366 pEAA367

Y4 pEAA368 pEAA369

YPS163 pEAA370 pEAA371

­­­­­­­­_____________________________________________________________________

MLH1 and PMS1 plasmids were derived from pEAA213 (S288c MLH1, LEU2, ARSH4,

CEN6) and pEAA238 (S288c PMS1, HIS3, ARSH4, CEN6), respectively.
